# Experimental study on the shear performance of an anchor cable with a C-shaped tube anchored flat structural plane

**DOI:** 10.1038/s41598-023-28105-1

**Published:** 2023-01-16

**Authors:** Xiao Tong, Renliang Shan, Nan Liu, Dong Liu, Yonghui Wei

**Affiliations:** School of Mechanics and Civil Engineering, China University of Mining and Technology (Beijing), Beijing, 100083 China

**Keywords:** Civil engineering, Fossil fuels

## Abstract

To analyse the influence of normal stress (*σ*_n_) and steel tube strength on an anchor cable with a C-shaped tube (ACC), we selected Q235 steel tubes and Q345 steel tubes as representative ACCs and carried out double shear tests of ACC-reinforced jointed rock masses. Based on the test results, the influence of the steel tube strength on the ACC axial force and shear force under different normal stresses, the characteristics of the shear force-shear displacement curve of the anchored flat structural plane (FSP) in the rock mass, and the ACC failure mode and contribution to the anchored concrete surface shear strength were studied. The test results show that under 2 ~ 10 MPa of *σ*_n_, the failure angle varies between 28° and 40° due to the bending of the ACC near the structural plane and increases with increasing *σ*_n_. Compared with Q235-ACC, Q345-ACC contributes more to the shear strength of the structural plane and can better exert its axial force when resisting the lateral shearing action of the structural plane. Additionally, we proved that *σ*_n_ is a main factor affecting the shear stiffness of the structural plane and that the Q345 C-shaped tube effectively improves the shear stiffness of an ACC-reinforced jointed rock mass and can more fully mobilize the anchor cable during shearing ductility in the tangential direction compared to the performance of the Q235 C-shaped tube. The research results can provide a reference for the further application of ACCs to roadways.

## Introduction

Due to long-term geological tectonic movement, a large number of structural planes form in a rock mass, and its mechanical properties are very complex and changeable. Considerable engineering practice has shown that the instability and damage of underground coal mine roadways are mainly caused by the dislocation and slippage of rock strata along a structural plane. Rock bolts and anchor cables are the main supporting materials to ensure the stability of the surrounding rock in coal mine roadways. Compared with the rock bolt, the anchor cable can exert a larger preload and can be anchored to stable deep surrounding rock. A better understanding of the anchor cable load transfer mechanism is beneficial to the development of anchor cable support technology. Scholars at home and abroad have performed a considerable amount of research on anchor cables through indoor experiments^1–5^ numerical simulations^6–8^, and anchoring theory^9–12^. These studies have helped to improve our understanding of the load transfer mechanism of anchor cables.

A laboratory test is a necessary method to study the load transfer mechanism between a rock mass and an anchor cable. Studying the tensile and shear mechanical properties of anchor cables is beneficial to the design of roadway support schemes. However, most indoor tests focus on the tensile properties of the anchor cables while ignoring the shear behaviour of the anchor cables^13–15^. In fact, field engineering has shown that the failure of anchor cables in underground mining projects is often caused by a combination of tensile and shear forces. Most anchor cable shear load studies have been conducted within the last 10–15 years. For example, Aziz et al.^3,16–18^ conducted a series of double shear tests on various types of anchor cables, and they found that the shear strength of the anchor cables was affected by the surface profile of the anchor cables. Li et al.^19,20^obtained the contribution of the bolt stiffness to the shear strength of the joint surface through double shear testing. Mirzaghorbanali et al.^21^conducted shear tests on anchor cables in a frictionless rock mass between joints and found that the peak shear force and corresponding shear displacement of the anchor cables decreased with increasing pretension. Li et al.^22–24^systematically studied the tensile and shear behaviour of rock bolts, which was helpful for understanding the performance of rock bolts under different loading conditions. These studies have greatly promoted the further development of anchor cables. However, these studies still had shortcomings. First, after excavation of the roadway, under the action of geostress and structural stress, there is normal stress on the structural surface itself. However, the influence of the initial normal stress is not considered in these reports. Second, current studies on the tensile and shear properties of rock bolts or cables mainly focus on the anchored section of the bolt or cable. However, the breakage of anchor cables in Chinese coal mines has occurred in the unanchored section. Shan Renliang et al.^25^developed a new type of anchor cable with a C-shaped tube (ACC), which can effectively solve the problem of breakage of the unanchored section of the anchor cable.

In this study, the shear behaviour of ACCs with different strengths under the condition of initial normal stress on the structural plane is systematically studied. One of the ACCs uses a Q235 steel tube (Q235-ACC), and the other uses a Q345 steel tube (Q345-ACC). This paper first introduces the concept and development of the ACC structure, and then carries out indoor shear tests of Q235-ACC and Q345-ACC under different normal stress conditions. The shear force-shear displacement curve characteristics of the anchored jointed rock mass and the ACC failure mode, shear stiffness and its shear contribution are analysed and compared to provide a basis for the application of ACCs in actual engineering. Finally, prospects are discussed.

## ACC structure introduction

### Introduction to the ACC

The ACC structure design concept is to improve and optimize the stress state of the free section of the anchor cable when it is applied in the field. While the anchor cable has a high axial bearing capacity, it can prevent the free section from being dislocated by rock formations and rock blocks squeezed into the hole. Fracture is caused by internal and other lateral actions.

**Figure 1:**
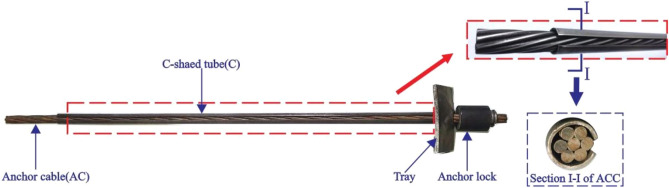
Structural composition of the ACC.

**Figure 2:**
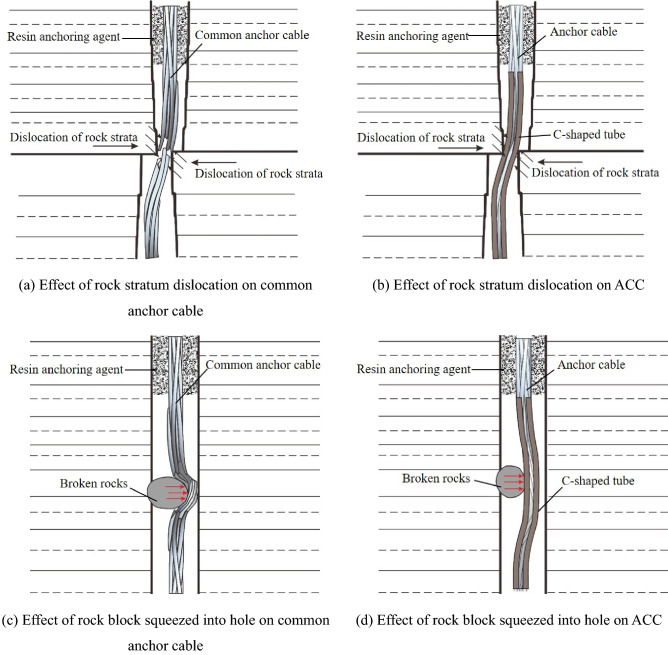
Schematic diagram of action mechanism of common anchor cable support and ACC support.

The ACC consists of an anchor cable, a C-shaped tube, an anchor lock and a tray. The structure and working principle of the ACC are shown in Figs. 1 and 2. As shown in Fig. 2, compared with common anchor cables, the ACC has a higher shear resistance and shear direction ductility because the C-shaped tube in the ACC can effectively protect the anchor cable, thereby effectively reducing the concentrated stress near the joint surface. The stress state of the anchor cable is improved by the friction between the tube and the anchor cable, thereby fully mobilizing the ductility of the anchor cable in the shearing direction; thus, the anchor cable is not easily broken by shearing.

### Method for determining the length of the C-shaped tube

Before support design, the surrounding rock structure along the roadway will be evaluated, the development of cracks (average depth) will be analysed, and the rock quality designation (*RQD)* index will be determined by core sampling. The length of the C-shaped tube is set according to the evaluation results of the surrounding rock structure. It is not limited to a fixed length. The control of the main fracture of such a rock mass is obtained by forming a reinforced arch structure through high pretension force, giving full play to the self-supporting capacity of the surrounding rock, and improving the stability of the surrounding rock, and only an anchor cable can meet this requirement. Thus, we used the C-shaped tube to solve the issue that the shear performance of the anchor cable will be reduced under a high preload. At the same time, a large number of on-site investigations have shown that the fracture depth appropriate for anchor cables and bolts is basically within 2 m of the surface of the roadway. Therefore, the C-shaped tube in the project is generally recommended to be 2 m long.

## Experimental method

### Test scheme

To study the influence of ACCs with different strengths on the shear strength of anchored jointed rock masses, Q235-ACC and Q345-ACC were taken as the research objects, and double shear tests of jointed rock masses under different normal stresses were carried out. In the testing, four normal stresses of the structural surface were set: 2, 4, 6, and 10 MPa. The test scheme is shown in Table 1, and the basic mechanical parameters of the C-shaped tube are shown in Table 2.

**Table 1:** Test scheme.

Product name	ACC properties					Pretension load (kN)	Normal stress (MPa)
	Cable *φ*(mm)	Peak tensile load (kN)	Cable elongation	Cable cross-section	C-shaped tube model		
Q235-ACC	21.6	531	7.1	7 wire	Q235	200	2,4,6,10
Q345-ACC	21.6	531	7.1	7 wire	Q345	200	2,4,6,10

**Table 2:** Basic mechanical parameters of the two tubes.

Material of tube	Outer diameter (mm)	Thickness (mm)	Width of the slot (mm)	Yield strength (MPa)	Tensile strength (MPa)	Elongation (%)
Q235 steel	28	2	8	300	415	36
Q345 steel	28	2	8	385	540	25.5

### Test loading system

**Figure 3:**
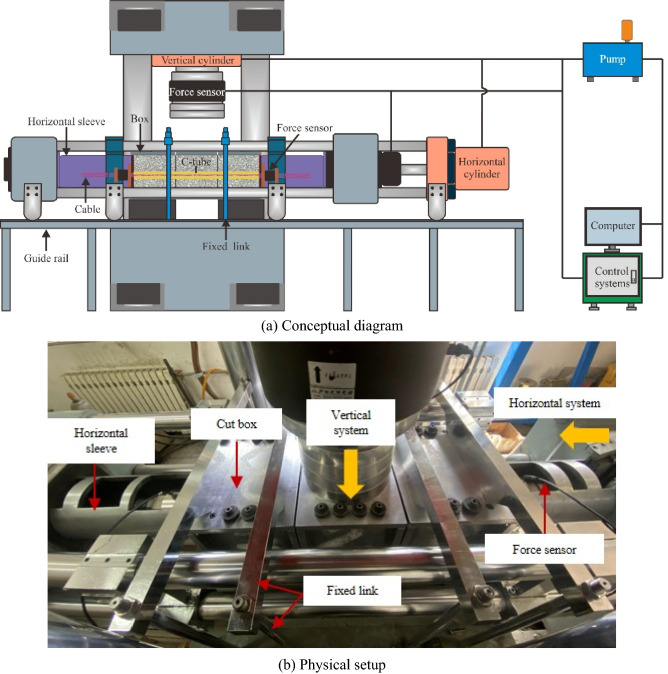
Large shearing equipment.

We used a modified large-scale double shear device to apply the target normal stress to the structural surface. The system is mainly composed of three parts: a loading system, a force‒displacement monitoring system and a loading control system, as shown in Fig. 3.

We applied normal stress to the structural surface through a horizontal sleeve inserted before the test, the normal loading rate was set to 0.1 kN/s, and the change value of the initial force per unit time was set with a computer. After the initial force value was stabilized, the vertical shear displacement was applied to the intermediate shear box using the displacement control method, and the shear displacement rate was set to 2 mm/min. The test was considered over when the ACC broke or the shear displacement reached 200 mm (the maximum displacement of the equipment). The monitoring system simultaneously recorded the shear force, axial force and shear displacement in real time. After the test, the specimen was removed from the shear box to analyse the failure characteristics of the ACC and the structural surface.

### Sample preparation

Notably, for the convenience of fabrication, a flat and unfilled structural surface was used in this test. Its mechanical properties were simple, and through the anchorage model test of the flat structural surface, the different effects of the anchorage structural surface in the shearing process can be clarified.

**Figure 4:**
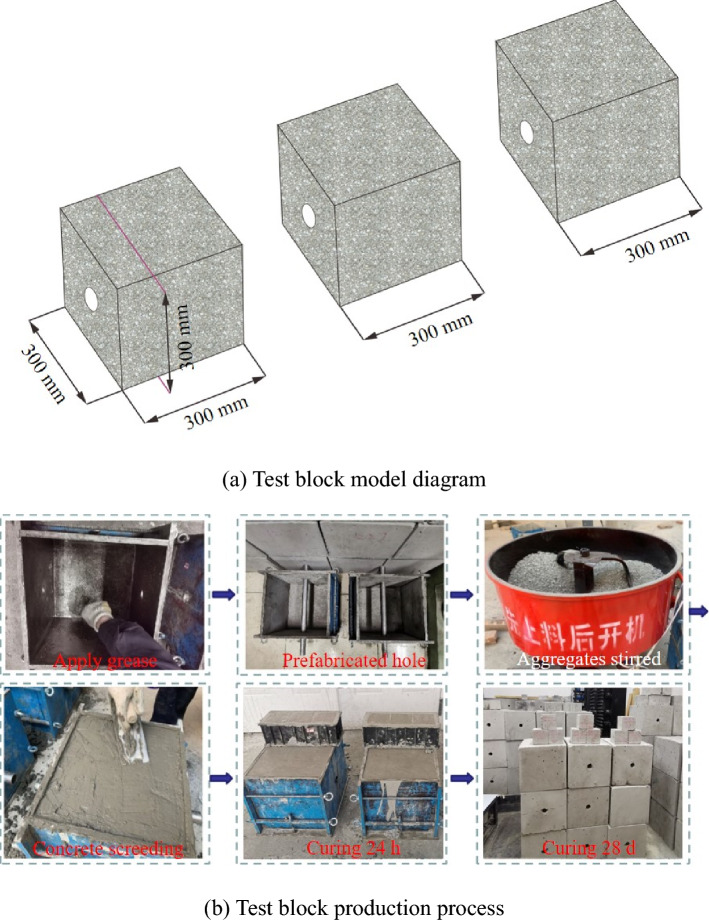
Dimensions and production process of anchored jointed rock mass model specimens.

The jointed rock mass model used in the test is shown in Fig. 4a, and the simulated material of the rock mass is made of concrete. Three concrete blocks were used in each set of experiments, each with a size of 300 mm × 300 mm × 300 mm. For the specific production process of concrete blocks in reference^26^, we mixed water, C42.5 cement, fine sand and stones in a ratio of 1:2:4:4. First, we poured the mixed aggregate into the steel mould and pre-embedded a steel pipe with a diameter of 32 mm in the mould to create the ACC hole. At the same time, we placed the same batch of concrete into a 100 mm × 100 mm × 100 mm standard test box to determine the uniaxial compressive strength (UCS) of the concrete block. The vibration-compacted and uniform aggregates were rested for 24 h, the moulds were removed, and the concrete blocks were placed in a maintenance room. Notably, to give full play to the mechanical and physical properties of the concrete blocks, 28 days of curing of all the concrete blocks were completed before testing. The production process of the concrete blocks is shown in Fig. 4b. We measured the average UCS of the concrete blocks to be approximately 41 MPa by uniaxial compression tests.

## Test results and analysis

### Deflection and failure characteristics of a rock mass with an anchored flat structural plane (FSP)

**Figure 5:**
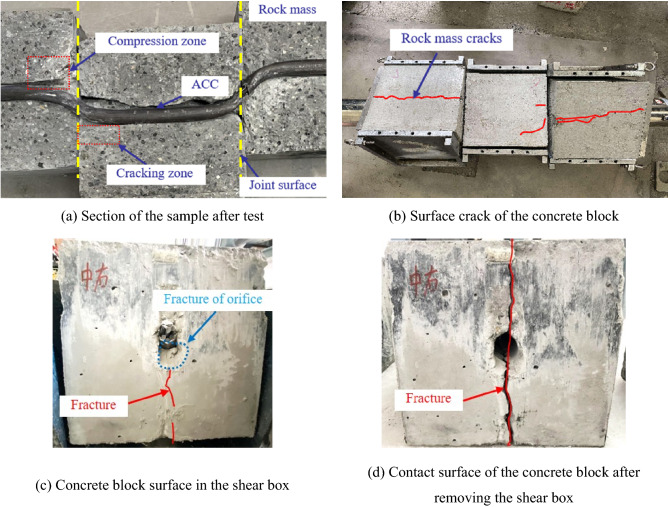
Failure characteristics of the ACC-reinforced jointed rock mass after testing.

Due to the combined effect of external loads and compression of the surrounding rock, when a bolt or cable is subjected to lateral shearing at a rock mass structural plane or discontinuity, a certain lateral deformation will inevitably occur^4,19^. After shear testing, analysing the failure characteristics of the rock mass and ACC is particularly important to understand the mechanism of action of the ACC-reinforced structural plane. Figure 5 shows the deformation and failure of a rock mass and the corresponding ACC after a shear test. Figure 5a indicates that the ACC underwent obvious shear deformation near the structural surface that results in a certain curvature. There are compression zones and cracking zones in the rock mass on both sides of the structural plane. In the compression zone, the contact of the ACC and hole wall produced compressive stress so that the rock mass in this area was crushed, resulting in an obvious wear area with a white colour. In the cracking zone, the rock mass also suffered a certain degree of damage. During the test loading process, since the borehole was squeezed by the ACC, obvious stress concentrations occurred at the orifice of the rock mass, and we believe that this phenomenon caused the concrete block to crack (see Fig. 5b). By observing Fig. 5c,d, we found that the surface cracks of the concrete blocks left in the shear box were small and not completely continuous, which indicates that the shear box can effectively restrain the failure of the concrete blocks. The shear box can effectively transfer the load to the concrete block and avoid the loss of bearing capacity caused by the complete crushing of the concrete block before the ACC breaks. In addition, we also observed that the lower part of the orifice of the concrete block was significantly broken due to the compression of the ACC (see Fig. 5c).

**Figure 6:**
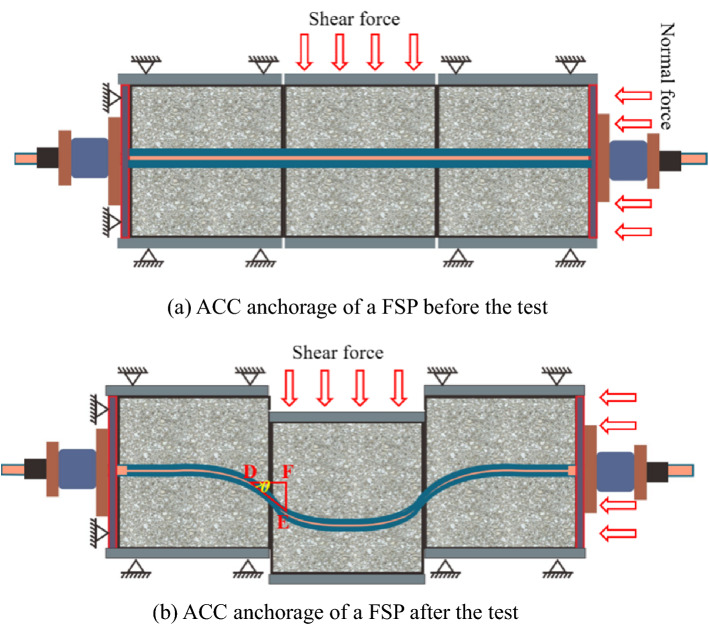
Diagram of the ACC anchorage of a FSP before and after testing.

During the shear loading process of the ACC-reinforced jointed rock mass, both tensile and shear forces were applied to the ACC. As shown in Fig. 6, in addition to the axial tensile deformation, near the structural plane, the ACC underwent bending deformation. When the bending moment on the ACC section reached its plastic limit bending moment, the ACC rotated, and finally, a pair of obliquely symmetrical plastic hinges formed on both sides of the structural plane^21^. To further analyse the deformation and failure characteristics of the ACC after the test, the ACC was removed after the concrete block was broken. We statistically analysed the geometric parameters of the ACC in the structural plane, such as the deformation length DE, vertical member length FE, horizontal member length DF and plastic hinge angle *θ*.

**Figure 7:**
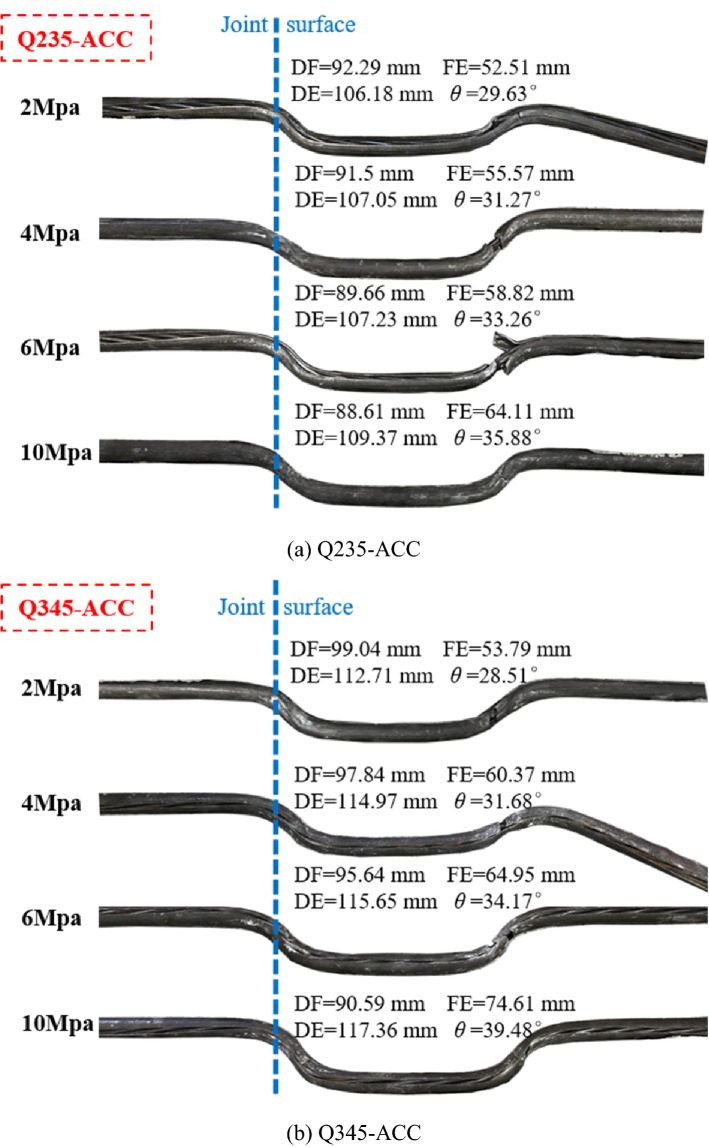
Photographs of the ACC failures and deformations.

**Figure 8:**
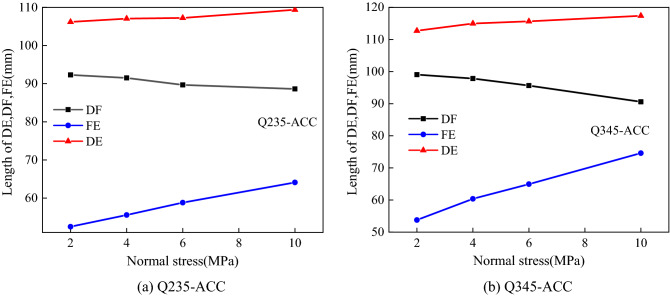
ACC deformation length DE, its horizontal component DF, and its vertical component FE.

**Figure 9:**
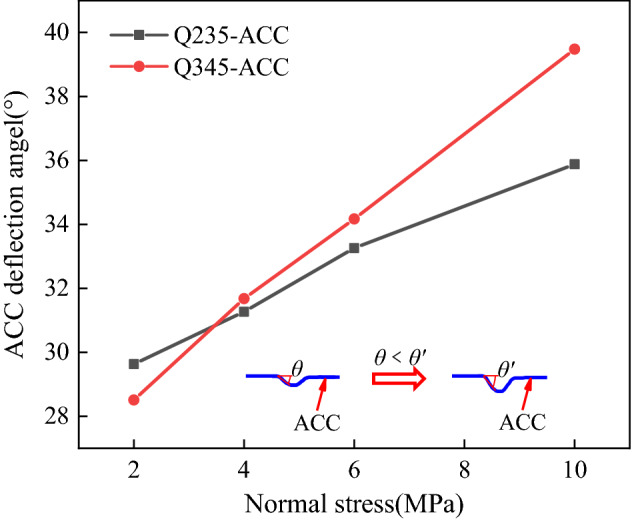
Relationship between the plastic hinge angle θ of the ACC and *σ*_n_.

Figure 7 shows that the Q235-ACC and Q345-ACC specimens under different *σ*_n_ were deformed and fractured after anchoring the FSP, and the parameters related to the plastic hinge of the ACC were measured and counted. The deformation length (DE) of Q235-ACC after breaking was between 106.18 and 109.37 mm, while the deformation length of Q345-ACC was greater than that of Q235-ACC, and its DE was between 112.71 and 117.36 mm. As shown in Fig. 8, under different *σ*_n_ conditions, the deformation length DE and its vertical component (FE) of all ACCs increased with increasing *σ*_n_. Its horizontal component (DF) decreased with increasing *σ*_n_. Figure 9 shows that the plastic hinge angle *θ* of Q235-ACC and Q345-ACC under 2 MPa, 4 MPa, 6 MPa and 10 MPa *σ*_n_ was between 28° and 40°. The plastic hinge angle θ increased with increasing *σ*_n_ of the structural plane. This was mainly because the compressive stress between the structural faces of the ACC-connected concrete blocks increased with increasing *σ*_n_. This caused the ACC near the structural surface to bear a large bending moment *M* during bending deformation, which in turn led to an increase in the plastic hinge angle *θ*. In addition, we found that the ACC will undergo a certain bending deformation under shearing action until it breaks. Therefore, we believe that it was precisely because of the increase in *σ*_n_ of the structural plane that the load applied to the ACC bending section increased, which in turn produced a larger bending deformation effect.

### Shear force–shear displacement

**Figure 10:**
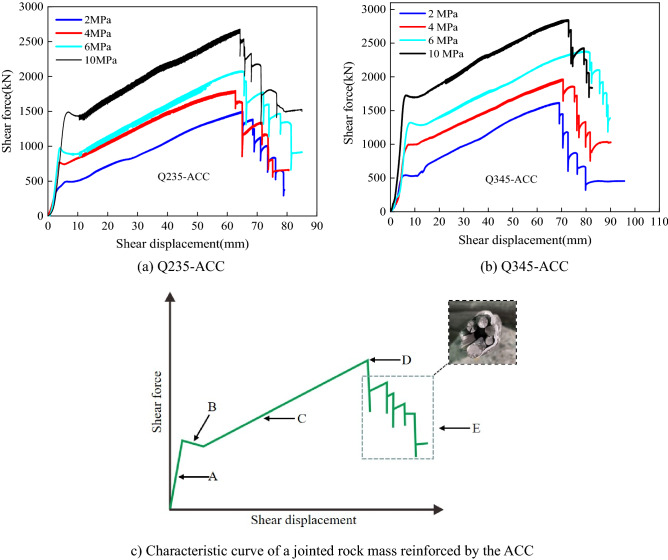
Shear force–shear displacement curves of the ACC-reinforced jointed rock masses.

Figure 10a,b show the shear force-shear displacement curves of Q235-ACC and Q345-ACC under four *σ*_n_, and the shear force shown in the figure is the total shear force of the system. We believe that this is due to the combination of shear forces generated by the lateral action of the ACC and shear forces provided by the system overcoming the frictional resistance between the surfaces of the concrete block structure. That is, the shear strength of the jointed rock mass in the test mainly comes from two parts: the frictional resistance between the structural planes and the shear resistance provided by the ACC. Figure 10c shows the shear force-shear displacement characteristic curve of the ACC-reinforced jointed rock mass. We divided the curve into the following five stages:

A: *Elastic deformation* During this phase, the relationship between the shear force and shear displacement can be considered linear. The shear force increases rapidly with increasing shear displacement. The resistance between the structural planes is caused by *σ*_n_ and the initial pretension of the anchor cable.
B: *Rock crushing* At this stage, transverse fractures are created in the jointed rock mass, and the force‒displacement curve drops suddenly.
C: *Yield stage* At this stage, the force acting on the anchored jointed rock mass is mainly borne by the ACC. The ACC exerts its "dowel effect", thereby resisting the relative deformation between the structural faces. In addition, shear deformation is still resisted by the friction between structural surfaces.
D: *Peak shearing load* After the yield stage, the peak load is achieved at a certain shear displacement.
E: *Shearing rupture* At this stage, the shear force suddenly decreases, the ACC breaks, and the downwards trend of the curve is "stepped". This is because the forces of the seven steel strands in the ACC are different, so the steel strands are broken one by one in the test.

### Shear stiffness

Shear stiffness is an important parameter reflecting the shear deformation properties of structural surfaces. In shear tests, shear stiffness is affected by various factors, such as concrete strength, joint friction coefficient, and pretension. Jalalifar^2^ attributed the change in the shear stiffness of bolt joints mainly to the magnitude of the bolt modulus. Yang et al.^5^ and Li et al.^20^ conducted shear tests and believed that the pretension of the anchor cable had a significant effect on the initial shear stiffness of the joint, but they did not consider the effect of *σ*_n_ of the structural surface on the shear stiffness. Subsequently, Shan et al.^27^ applied an initial normal stress to the structural surface in the shear test. These investigators found that with increasing initial normal stress of the structural surface, the average shear stiffness of the anchor cable and ACC fluctuated randomly within a certain range, with no obvious regularity. We believe that the reason why this study does not find an obvious regularity may be that the difference among the *σ*_n_ values tested in this study is too small and that the number of tests is insufficient.

**Figure 11:**
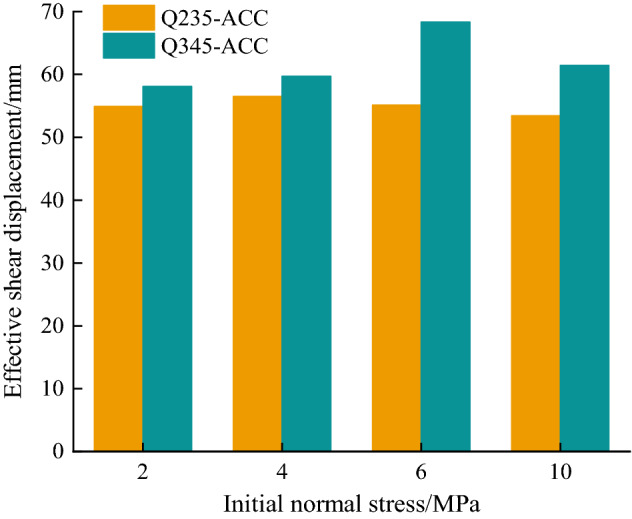
Comparison of the effective shear displacement.

To clarify the influence of the *σ*_n_ of the structural plane on the shear stiffness, the slope of the shear force-shear displacement of the structural plane is defined as the shear stiffness of the structural plane. Notably, to eliminate the impact of specimen rupture on the shear displacement, we used the concept of effective shear displacement for analysis; that is, we only considered the shear displacement generated when the structural plane shears the ACC. Figure 11 shows the effective shear displacements of Q235-ACC and Q345-ACC under different initial normal stresses.

The curves in Fig. 10a,b show that the shear stiffness of the structural plane is constantly changing during the test. To facilitate the analysis, the average shear stiffness of the structural surface within the effective shear displacement was calculated in this study, and the calculation results are shown in Table 3. Table 3 shows that with increasing *σ*_n_, the average shear stiffness of Q235-ACC and Q345-ACC also increases. This shows that when *σ*_n_ is in the range of 2 ~ 10 MPa, *σ*_n_ is one of the main factors affecting the shear stiffness of the structural plane. In addition, the shear stiffness of Q345-ACC is higher than that of Q235-ACC at the same *σ*_n_. Consequently, compared to Q235 C-shaped tubes, the Q345 C-shaped tube can significantly improve the shear stiffness of jointed rock masses reinforced with ACCs.

**Table 3:** Shear stiffnesses of ACC-reinforced jointed rock masses (ACC loaded to failure).

Bolt type	Normal stress (MPa)	Average shear stiffness (kN/mm)
Q235-ACC	2	4.64
Q235-ACC	4	6.71
Q235-ACC	6	8.19
Q235-ACC	10	11.7
Q345-ACC	2	4.97
Q345-ACC	4	8.4
Q345-ACC	6	9.38
Q345-ACC	10	13.84

### Shear strength parameters of rock masses with ACC-reinforced FSPs

**Figure 12:**
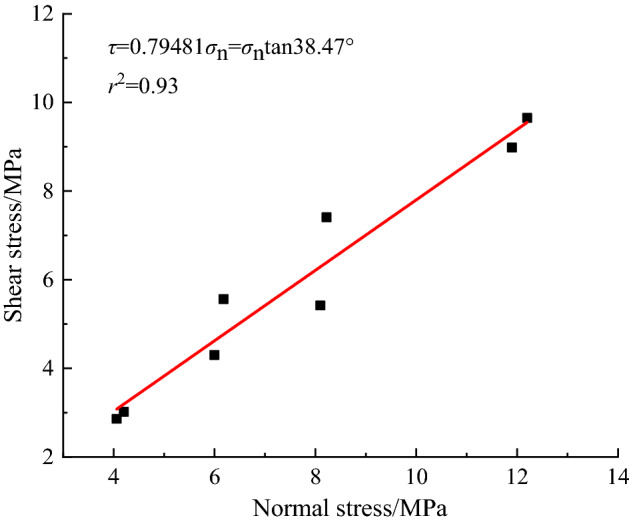
Fitting curve of the shear strengths of the tested ACC-reinforced jointed rock masses.

The shear strength of a structural plane can be assumed to follow the Mohr‒Coulomb strength criterion. Because a FSP in this experiment is the contact surface between two concrete blocks, the cohesion *c* of the structural surface is approximately 0 MPa. Shan et al.^27^ used the same equipment as used in this test to conduct an indoor test on the shear mechanical properties of a pretension anchor cable and an ACC. Their research shows that the shear force of the structural surface can just overcome the frictional resistance according to the shear test (that is, when the structural surfaces are sliding relative to each other, but the concrete blocks do not exhibit shear effects on the ACC). Combined with the axial force at this time, we calculated the *σ*_n_ and shear stress acting on each structural surface. Then, we analysed the shear strength parameters of the simulated samples of the ACC-reinforced jointed rock masses by the least squares method according to the *σ*_n_ and shear stress of each group just overcoming the friction of the structural surface in the test. The analysis curve is shown in Fig. 12.

1 $$ \tau = \sigma_{n} \tan \varphi $$

where *τ* is the shear strength (MPa), *σ*_n_ is the initial normal stress (MPa), and *φ* is the internal friction angle (°).

By fitting, the constitutive relation of the structural plane approximately satisfies:

2 $$ \tau = 0.79481\;\sigma_{n} \;\;\;r^{2} = 0.93 $$

In addition, the internal friction coefficient is 0.79 according to the obtained shear strength parameters of the ACC-reinforced rock mass, so the friction angle of the structural surface was calculated to be 38.47° through the inverse trigonometric function.

### ACC contribution to joint shear strength

The shear contribution of bolts to jointed rock masses is an important issue in the study of anchored jointed rock masses. Grasselli^8^ and Jalalifar^28^ defined the contribution equation of a bolt to the joint shear strength according to test records and analysis:

3 $$ R = F_{s} - F_{0} \tan \varphi $$

where *R* is the contribution of the bolt to the shear strength of the joint (kN), *F*_s_ is the shear force of the rock mass anchored at the joint (kN), *F*_0_ is the initial normal force at the structural surface (kN), and *φ* is the joint friction angle (°).

Table 4 shows the calculation results of the shear resistance contribution of Q235-ACC and Q345-ACC to the FSP. The average values of the shear contribution *R* of Q235-ACC and Q345-ACC are 595.92 kN and 706.94 kN at *σ*_n_ of 2, 4, 6 and 10 MPa. The shear contribution *R* of Q345-ACC is higher than that of Q235-ACC.

**Table 4:** Statistics of the ACC contribution and the axial and shear forces at failure.

Type	Normal stress (MPa)	ACC contribution, *R* (kN)	Average *R* (kN)	Axial force, *N*_0_ (kN)	Shear force, *Q*_0_ (kN)	*Q*_0_/*N*_0_	Average *Q*_0_/*N*_0_
Q235-ACC	2	597.97	595.92	447.14	142.92	0.32	0.34
	4	596.95		444.77	144.75	0.36	
	6	596.92		444.23	145.17	0.33	
	10	591.87		439.59	148.67	0.34	
Q345-ACC	2	664.97	706.94	508.71	134.12	0.26	0.28
	4	695.95		524.04	153.09	0.29	
	6	759.93		560.3	202.01	0.36	
	10	706.88		539.7	122.91	0.23	

**Figure 13:**
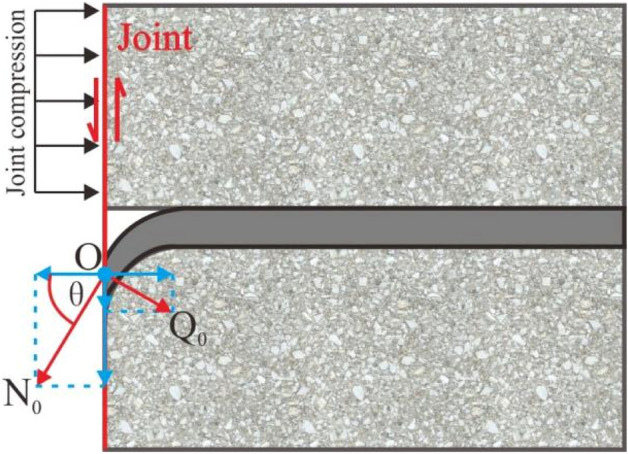
Schematic diagram of the ACC reinforcement of a jointed rock mass.

Previous studies have shown that the shear contribution of bolts to the structural plane is mainly manifested in two aspects, namely, the direct contribution *R*_d_ and the indirect contribution *R*_i_. The direct contribution *R*_d_ is related to the parallel components of the axial force *N*_0_ and the shear force *Q*_0_ on the structural surface, while the indirect contribution *R*_i_ is related to the vertical component of the axial force *N*_0_ and the shear force *Q*_0_ on the structural surface, as shown in Fig. 13. The calculation equations of the direct contribution *R*_d_, indirect contribution *R*_i_, axial force *N*_0_ and shear force *Q*_0_ are as follows^4,20,29^:

4 $$ R = R_{d} + R_{i} $$

5 $$ R_{d} = \left( {N_{0} \cos \theta - Q_{0} \sin \theta } \right)\tan \varphi $$

6 $$ R_{i} = N_{0} \sin \theta + Q_{0} \cos \theta $$

where *R* is the contribution of the anchor to the shear strength of the structural plane (kN); *R*_d_ is the direct contribution (kN); *R*_i_ is the indirect contribution (kN); *N*_0_ and *Q*_0_ are the axial force and shear force at the structural surface when the bolt is broken (kN), respectively; and *θ* and *φ* are the bolt deflection angle and joint friction angle (°), respectively.

Dight^30^ proposed an analytical equation to predict the shear failure of a bolt; then, the axial force *N*_0_ and the shear force *Q*_0_ should satisfy the following equations:

7 $$ \left( {\frac{{N_{0} }}{{N_{f} }}} \right)^{2} + \left( {\frac{{Q_{0} }}{{Q_{f} }}} \right)^{2} = 1 $$

8 $$ N_{f} = A_{b} \sigma_{f} ,\;\;\,Q_{f} = A_{b} \tau_{f} $$

where *N*_f_ and *Q*_f_ are the ultimate tensile and shear forces of the bolt (kN), respectively; *σ*_f_ and *τ*_f_ are the tensile strength and shear strength of the bolt (MPa), respectively; and *A*_b_ is the cross-sectional area of the bolt (mm^2^).

According to the Tresca criterion,

9 $$ \tau_{f} = \frac{{\sigma_{f} }}{2} $$

Combined with Eqs. (3), (4), (5), (6), (7), (8), the shear contribution *R*, ACC deflection angle *θ*, joint friction angle *φ*, ACC tensile strength *N*_f_, shear strength *Q*_f_, and cross-sectional area of the ACC were obtained through laboratory test results. According to Eqs. (3) and (6), we calculated the axial force *N*_0_ and shear force *Q*_0_ of Q235-ACC and Q345-ACC under different *σ*_n_ values. The calculation results are shown in Table 4. The shear force when the ACC breaks at the structural surface is much lower than the axial force. A higher support resistance in the ACC-reinforced jointed rock mass is needed to achieve a better support effect. Thus, the ACC should bear a higher axial force compared to the shear force. According to the values of the axial force *N*_0_ and the shear force *Q*_0_, the *Q*_0_/*N*_0_ ratio of Q234-ACC and Q345-ACC under different *σ*_n_ values are 0.34 and 0.28, respectively. This indicates that compared with Q345-ACC, Q234-ACC is subjected to a higher shear force when breaking, so Q234-ACC exhibits less deformation when breaking. However, when resisting transverse shear deformation, Q345-ACC can better exert its axial force. This shows that Q345-ACC can bear more shear force than Q234-ACC when strengthening a jointed rock mass, which proves that the Q345 C-shaped tube can better improve the supporting effect of an ACC-reinforced jointed rock mass.

## Prospects

Due to research limitations our work on the shear mechanical properties of ACC-reinforced red joint surfaces is still relatively shallow, and further research is necessary:

1. In this test, the simulated joints are unfilled, with coincident fracture surfaces and no tiplines. However, most natural cracks have fillers, have surfaces that do not coincide, are of finite size, and are in a complex three-dimensional stress state. In future research, it is necessary to carry out experimental analysis and research on structural surfaces with different roughnesses, mainly under different normal stress conditions and different ACCs, and the relationship between the mechanical properties of structural surface materials. The calculation formula of the shear contribution parameters of ACCs to structural planes should be further modified and improved so that it has better universality.
2. In-depth research on ACC anchoring jointed rock masses should be performed. In future work, it will be necessary to improve the identification of the mechanical mechanism controlling ACCs and conduct numerical simulation analysis on the mechanical properties of ACC-reinforced jointed rock masses. In-depth research on the mechanical properties of ACC-reinforced jointed rock masses can be carried out by means of theoretical derivation, laboratory tests and numerical simulation.
3. When a jointed rock mass contains three or more groups of random structural planes, the engineering mechanical properties of the jointed rock mass tend to be isotropic. However, due to the strong probability and statistical characteristics of these structural surfaces, when considering different shear directions, the obtained shear paths or shear failure surfaces will be different. Therefore, in the next step of research on the anchoring mechanism of prestressed ACC, the probability distribution characteristics of random structural surfaces should be considered. The mechanical properties of prestressed ACC-reinforced jointed rock masses should be studied in depth by using the method of probability and statistical analysis.

## Conclusion

In this study, indoor testing of ACC-reinforced FSPs under different *σ*_n_ conditions was carried out by using a large shear instrument, the shear resistance of the ACC-anchored structural surface was investigated, and Q345-ACC and Q235-ACC control groups were used for comparison. From the experimental results, the main conclusions were as follows:

1. By analysing the failure characteristics of the ACC and the jointed rock mass, we found that local shear deformation occurred in the ACC near the structural plane, and the ACC and the concrete blocks near the structural plane formed compression zones and cracking zones. In addition, a pair of obliquely symmetrical plastic hinges were formed on both sides of the structural plane. We also determined that the plastic hinge angle *θ* increases with increasing *σ*_n_ acting on the structural plane.
2. The shear force-shear displacement curve of the ACC-reinforced FSPs can be divided into four stages. The shear strength of a jointed rock mass is mainly derived from the frictional resistance between the structural planes and the shear resistance provided by the ACC.
3. We found that Q345-ACC had a higher shear strength contribution to FSP than Q235-ACC. Compared with Q235-ACC, Q345-ACC can better exert its axial force when resisting the lateral shear of the structural plane. This shows that Q345-ACC can bear more shear force than Q234-ACC when strengthening a jointed rock mass, which proves that the Q345 C-shaped tube can better improve the supporting effect of an ACC strengthening a jointed rock mass.
4. This paper proves that *σ*_n_ is one of the main factors affecting the shear stiffness of the structural plane, and compared with the Q235 C-shaped tube, the Q345 C-shaped tube can effectively improve the shear stiffness of an ACC-reinforced jointed rock mass.

## Data Availability

The datasets used and analyzed during the current study are available from the corresponding author on reasonable request.
